# Perceiving Nasal Patency through Mucosal Cooling Rather than Air Temperature or Nasal Resistance

**DOI:** 10.1371/journal.pone.0024618

**Published:** 2011-10-13

**Authors:** Kai Zhao, Kara Blacker, Yuehao Luo, Bruce Bryant, Jianbo Jiang

**Affiliations:** Monell Chemical Senses Center, Philadelphia, Pennsylvania, United States of America; Hospital of the University of Pennsylvania, United States of America

## Abstract

Adequate perception of nasal airflow (i.e., nasal patency) is an important consideration for patients with nasal sinus diseases. The perception of a lack of nasal patency becomes the primary symptom that drives these patients to seek medical treatment. However, clinical assessment of nasal patency remains a challenge because we lack objective measurements that correlate well with what patients perceive.The current study examined factors that may influence perceived patency, including air temperature, humidity, mucosal cooling, nasal resistance, and trigeminal sensitivity. Forty-four healthy subjects rated nasal patency while sampling air from three facial exposure boxes that were ventilated with untreated room air, cold air, and dry air, respectively. In all conditions, air temperature and relative humidity inside each box were recorded with sensors connected to a computer. Nasal resistance and minimum airway cross-sectional area (MCA) were measured using rhinomanometry and acoustic rhinometry, respectively. General trigeminal sensitivity was assessed through lateralization thresholds to butanol. No significant correlation was found between perceived patency and nasal resistance or MCA. In contrast, air temperature, humidity, and butanol threshold combined significantly contributed to the ratings of patency, with mucosal cooling (heat loss) being the most heavily weighted predictor. Air humidity significantly influences perceived patency, suggesting that mucosal cooling rather than air temperature alone provides the trigeminal sensation that results in perception of patency. The dynamic cooling between the airstream and the mucosal wall may be quantified experimentally or computationally and could potentially lead to a new clinical evaluation tool.

## Introduction

The subjective sensation of nasal airflow, or nasal patency, is of great importance to patients with nasal sinus disease. A perception of a lack of nasal patency is the primary symptom that drives these patients to seek medical treatment. However, this subjective perception often bears little relationship to the actual physical resistance to airflow in the nose. Objective evaluation tools, such as acoustic rhinometry, rhinomanometry, CT staging scores, and endoscopic examination, often poorly correlate with subjective patency [1], [2], even though these objective tools often correlate well with each other. Objectively assessing subjective perceptions of nasal patency remains a challenge.

It is generally believed that the perception of nasal patency involves nasal trigeminal activation by cool inspiratory air flow, possibly mediated by TRPM8 channels expressed in trigeminal C-fiber or A-δ fiber endings that innervate nasal epithelium [3]. Pharmacological modulation of trigeminal feedback has been shown to alter patency perception. For example, topical or oral application of menthol produces the illusion of decongestion and improved nasal airflow without actually altering nasal morphology [4]. In contrast, topical application of local anesthetics on the nasal epithelium results in an artificial sensation of nasal obstruction, presumably due to blockage of the trigeminal feedback [5].

Menthol was widely used as an ingredient in common cold medications, nasal spray, candy, chewing gum, and cigarettes long before its target receptor, the non-selective voltage-dependent cation channel TRPM8, was identified [6], [7]. It is now known that menthol and air temperature both shift TRPM8 voltage-activation dependency toward the physiological range, and their effects are additive [8]. Thus, when combined, menthol and cool air can greatly enhance TRM8 activation. The nonspecific cation channel TRPA1 on other neurons is activated at much colder (noxious) temperatures and is not affected by menthol. However, its role in the sensation of cooling is not clear.

Despite the plausible role of trigeminal cool receptor input in perception of nasal patency, factors that stimulate or modulate this signal remain unclear. Is air temperature the primary stimulus providing the sensation of nasal patency? One study found that air temperature modulates perceived patency [9], but subject exposures and air humidity were not well controlled. Also, no study has investigated whether variation in trigeminal sensitivity is a determinant of perceived patency. Such variation could predispose some individuals to experience heightened symptoms of nasal obstruction. Furthermore, a full analysis of the possible factors that may contribute, both independently and in combination, to the perception of nasal patency is lacking.

The current study aimed to identify physical and physiological factors contributing to the sensation of nasal patency. First, we set out to confirm that nasal patency is indeed modulated by air temperature (hypothesis 1). Next, we tested whether air humidity affects nasal patency, while holding air temperature constant. We hypothesized that if air humidity indeed significantly influences perceived patency, it would suggest that mucosal cooling, which is the combination of both conductive heat loss (driven by temperature gradient) and evaporative heat loss (driven by water vapor pressure gradient), is the actual underlying factor contributing to the sensation of nasal patency, rather than temperature alone (hypothesis 2). Finally, we examined whether other factors, such as general trigeminal sensitivity or nasal resistance, may also contribute as alternative or confounding factors (hypothesis 3). We believe that clinical assessment and improvement of subjective nasal patency will be enhanced by fully understanding the underlying mechanisms that influence the perception of patency.

## Methods

The study was conducted in an air-conditioned, well-ventilated testing room at the Monell Chemical Senses Center (Philadelphia, PA).

### Subjects

Forty-four healthy volunteers (24 females and 20 males) were recruited from the local population for this study. This study was approved by the University of Pennsylvania institutional review board. Written informed consent was obtained from all volunteers that participated in the study. Their ages ranged from 20 to 61 years, with a mean of 29, median of 25, and standard deviation of 9.4 years. All of the participants underwent medical history screening to exclude preexisting nasal sinus disease, and both acoustic rhinometry and rhinomanometry were performed on all subjects to objectively confirm the absence of severe nasal obstruction.

### Exposure setting

Three facial exposure boxes, 24×24×24 cm, with a volume of 13.8 L, were constructed of acrylic plates. Three different air sources were pumped into each of the boxes at the same flow rate (10 L/min): (a) untreated room air; (b) dry air, generated by passing room air through a Drierite (anhydrous calcium sulfate) column, which removed much of the moisture; and (c) cold air, generated by passing room air though a copper coil submerged in ice. We insulated the cold facial exposure box and its connecting tubes with foam padding (Reflectix, Inc., Markleville, IN) to reduce heat loss and placed ice packs inside to reduce the air temperature within the box. A triangle-shaped window, designed to allow insertion of the nose, was cut into each of the boxes, and its edges were covered with foam rubber to ensure a tight fit. Small fans (CPU cooling fan, powered by 12 V DC) were installed inside each box to enhance air mixing. We ensured that the airflow created by the fan and by the pump did not blow directly toward the subject. The whole system was turned on 10 min prior to testing, with the exposure window covered, to allow the boxes to reach equilibrium.

**Figure 1:**
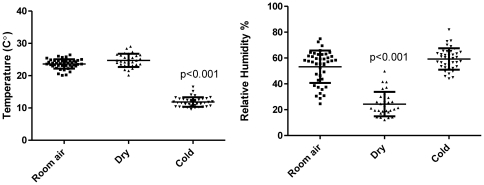
Temperature (left) and relative humidity (right) measurement during each subject exposure session. The temperature in the cold air exposure box was significantly lower than in the other exposure boxes, and the RH in the dry air exposure box was significantly lower than in the others (both p<0.01). The temperature difference between room air and dry air and the humidity difference between room air and cold air were both non-significant.

Temperature and relative humidity (RH) of the air inside the boxes and in the room were measured by a coupled thermometer and humidity sensor (SHT7x, Sensirion, Switzland) and recorded via USB connection to a PC (Figure 1). The sensor has an accuracy of ±1.8% for RH and ±0.3°C for temperature, with a response time less than 4 s. Recordings during subject exposures indicate that the air temperature of the cold air exposure box (mean ± SD, 11.8±1.5°C) was significantly lower than the two other conditions (room air box, 23.6±1.5°C; dry air box, 24.7±1.9°C; both p<0.001) and that the air humidity of the dry air box (26.8±12.2%) was significantly lower than the other two conditions (cold air box, 59.2±8.3%; room air box, 48.6±13.3%; both p<0.001). The temperature difference between room and dry air, and the humidity difference between room and cold air were both non-significant (Figure 1).

### Exposure procedure

**Figure 2:**
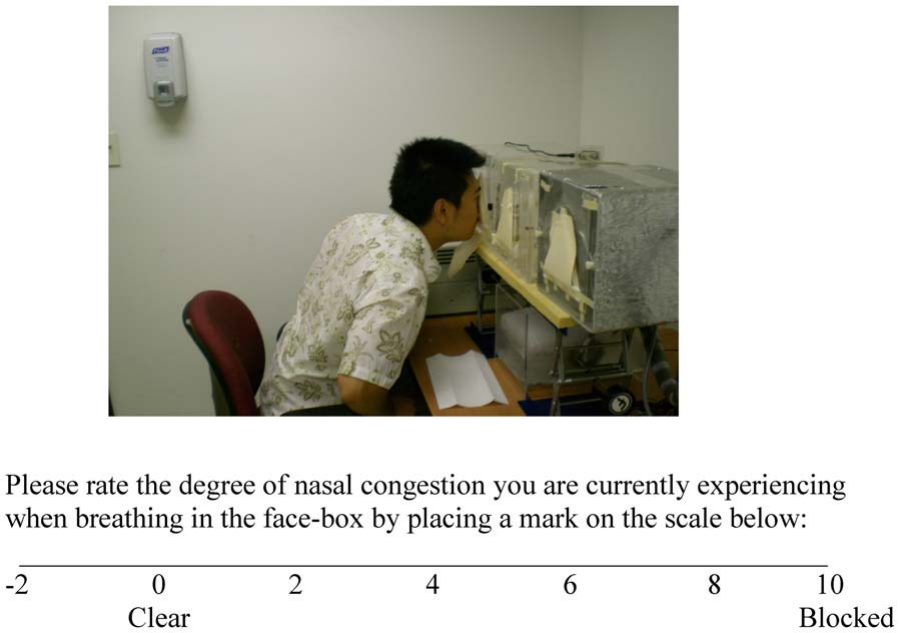
Air exposure boxes and the visual analog scale used in the study. The visual analogue scale includes a negative range to account for subjects that may rate their nasal patency as completely clear and then experience even less congestion in later exposures.

**Figure 3:**
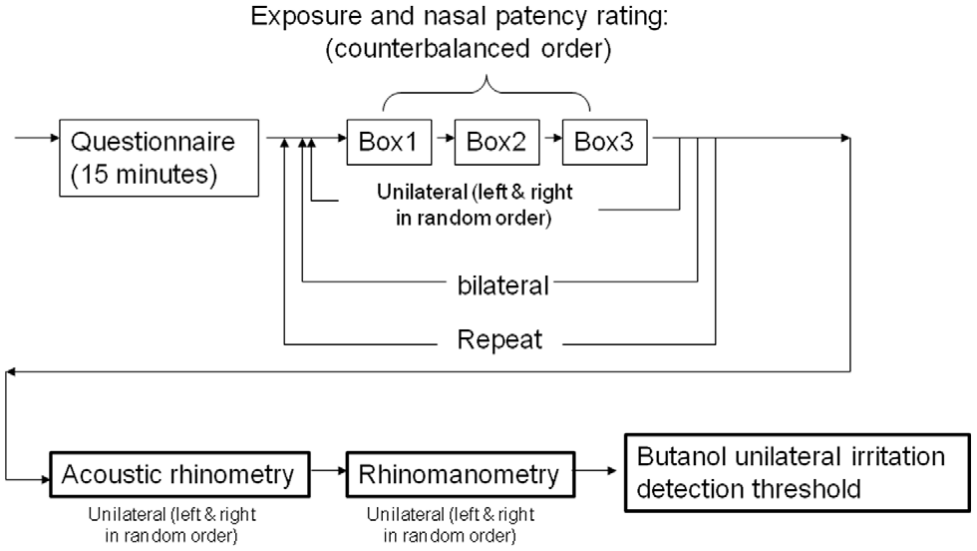
Flow chart of the test sequence for each participant: box exposure and patency rating (top), followed by rhinometry, rhinomanometry, and trigeminal assessment by butanol lateralization threshold (bottom).

Prior to testing, every subject was allowed 15 minutes to acclimatize to the room conditions while providing informed consent and filling out a medical history questionnaire. Subjects were then asked to sample the air in each box and then rate their perceptions of nasal congestion (perceived patency) using a visual analogue scale (Figure 2). The test sequence for each participant is illustrated in Figure 3 (top). Specifically, subjects sampled the air inside each of the three face boxes (untreated room air, cold air, and dry air) in counterbalanced order, with a 20-s interval between each sampling. For each sampling, they were instructed to remove the cover, insert their nose into the exposure window, and take only 1–3 breaths at their normal inspiratory depth, and then to rate their perception of nasal patency. Assuming an average tidal volume of 500 ml per breath for humans, we expected that the short exposure would not significantly modulate the condition of air inside the box, which was confirmed by the temperature and RH recordings made at baseline and during the subject exposures. We also assumed that a short (1–3 breath) exposure would not significantly alter the nasal mucosa swelling and sympathetic nervous system feedback of the subjects. Thus, the differences in ratings among the different conditions can be mostly attributed to the stimuli and perceptual mechanisms, rather to underlying mucosa structural changes, as has been reported for longer (>15 minute) exposures [10]. Bilateral exposure and ratings were performed first, followed by unilateral exposure and rating, during which, in random order, a foam nose plug was used to occlude the untested nostril. The whole procedure was then repeated, and the two ratings for each condition were averaged.

Because our subject cohort consisted of healthy individuals, it is likely that some subjects may rate their nasal patency as completely clear and then in later exposures experience even less nasal congestion. Thus, the visual analogue scale that subjects used to rate nasal patency was modified to include a negative range (see Figure 2).

Data from pilot trials showed that subjects would feel significantly more congested while breathing air in the test room compared with breathing air in the box ventilated with room air, a possible psychological effect. Thus, it is necessary to include the room air box as the control condition, rather than room air in the open room. Subjects were not informed of the condition of each box. They were informed that the air they would be sampling originated from the room and was completely safe.

### Objective rhinometry measurement

The cross-sectional area of the nasal cavity of each subject was assessed unilaterally by acoustic rhinometry (SRE21000, RhinoMetrics A/S, Denmark) as a function of the distance along the nasal passage from the nostril plane. The wave tube was attached to one nostril at a time; four readings were obtained and averaged from each nostril to ensure a reliable measurement. The minimum (narrowest) cross-sectional area (MCA) in the anterior 5 cm of nasal airway was collected.

The nasal resistance [11] of each subject during normal breathing was measured unilaterally by anterior rhinomanometry (SRE21000, RhinoMetrics A/S, Denmark). One nostril was occluded with an adhesive patch connected to a pressure transducer to measure nasopharyngeal pressure. Subjects were then requested to breathe quietly at a peak airflow rate slightly above 10 L/min through the other nostril into a pneumotachometer, where the airflow rate is measured simultaneously. The resistance is recorded as the flow rate at a predetermined pressure drop of 75 pascals between atmospheric pressure and the pressure in the nasopharynx, averaged over 7–8 breaths.

### Trigeminal detection thresholds

Unilateral irritation detection thresholds for butanol were obtained by using an objective, two-alternative, forced-choice, modified staircase method [12]. At each trial, the subject sniffed with both nostrils simultaneously from a pair of bottles: one contained a blank, and one contained appropriately diluted butanol. Subjects were then asked to identify which nostril received butanol. The butanol was diluted in a series of 15 binary dilution steps: the first step contained 50% butanol dissolved in mineral oil; the next step, 25% butanol; the next step, 12.5%; and so on. The blank was 10 ml of mineral oil. The pair of bottles, 280 ml in size, were both capped and connected to Teflon nosepieces, which, during the trial, were inserted into each nostril. The nosepieces were replaced and cleaned after each use. If a subject correctly identified the nostril receiving butanol in two consecutive trials, the next test concentration was decreased by one dilution step. If the subject was incorrect on any trial, the next test concentration was increased by a dilution step. The sequence ended after the subject had made at least five valid reversals in concentration level. The average of the final four was defined as the detection threshold. The test is based on the fact that nonirritating odorants cannot be lateralized and thus the lateralization threshold is a measure of nasal trigeminal sensitivity to volatile compounds that is independent of the chemical's effect on the olfactory system.

One important modification that we made to the above scheme in order to obtain unilateral thresholds is that left and right lateralization reversals are tracked separately, while the presentation order to either side remained random. For example, the dilution step presented to the left side in one trial depended only on the previous correct or incorrect identification in trials when the butanol was presented to the left side. Similarly, the reversal for the right side was determined only based on the previous trial when butanol was presented to the right side.

Butanol concentrations in the head space of each dilution bottle were measured and calibrated with gas chromatography and is reported in log parts per million (ppm).

### Hypotheses and data analysis:

The design of the experiments first aimed to determine the physical stimuli to nasal patency sensation. If sensation is modulated by air temperature (hypothesis 1), then we should find a significant change from baseline in exposure to the cold air box. However, exposure to the dry air box, which had the same temperature as the room air, may not elicit significant change from baseline if temperature is the sole dominant factor. On the other hand, if nasal patency sensation is modulated predominantly by air humidity, then we should find little difference in the effects from the cold air and room air boxes, because they had comparable humidity (see Figure 1). Two-tailed Wilcoxon matched (paired nonparametric) t-tests were used to examine this hypothesis. Holm–Bonferroni correction was applied to control for multiple comparison.

To test whether nasal patency sensation is mediated by nasal mucosal cooling (or heat loss) rather than by air temperature or humidity alone (hypothesis 2), we examined whether the computed total nasal mucosal heat loss (*q*) during breathing was a better predictor of nasal patency than temperature or RH alone, using Equation 1:
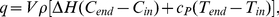
(1)where *V* is the inhaled air volume, assumed to be 500 ml, the average human tidal volume per breath; ρ is the air density (1.225 kg/m^3^); Δ*H* is enthalpy of water vaporization (2.4×10^6^ J/kg); *C*_end_ is air humidity at the nasal pharynx, in absolute units (0.0358 kg/m^3^) or 90% RH at 35°C; *C*_in_ and *T*_in_ are the recorded air temperature and humidity during each box exposure; *c*_P_ is the specific heat capacity of air (1006.43 J/kg·K); and *T*_end_ is air temperature at the nasal pharynx (35°C or 308.15 K). We assume that, regardless of the ambient air temperature and humidity, inspired air is always warmed and humidified up to 32°C (*T*_end_) and 90% relative humidity (*C*_end_) at the end of the nasal passage during breathing. Previous studies [13], [14] have indicated that the human nose is very efficient in warming and humidifying inhaled air during short exposures even under extreme ambient conditions. The total nasal heat loss (*q*), as the sum of conductive and evaporative heat loss, was then used as an independent variable while repeating correlation and multiple regression analyses.

The third hypothesis of this study examined whether factors such as lateralization thresholds to butanol (a stimulus widely used to measure general trigeminal sensitivity), nasal resistance, or minimum airway cross-section area (MCA), measured by rhinomanometry and acoustic rhinometry, respectively, also contributed as alternative or confounding factors in modulating perceived nasal patency. This hypothesis was tested using Spearman rank correlation and stepwise multiple regression. During stepwise regression, all the parameters, including air temperature and humidity, were input as independent variables, and nasal patency was the dependent variable. A forward regression scheme (F>1 for an independent variable to enter, F<0.5 to remove, steps = 5) was used to automatically pick a subset of the most significant variables from the pool of independent variables.

### Data exclusion

**Figure 4:**
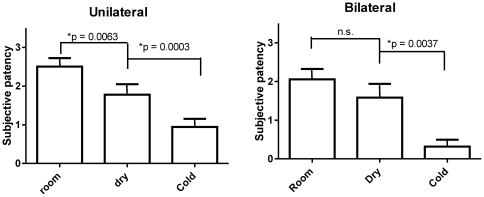
Bilateral and unilateral subjective patency ratings and standard errors in room air, dry air, and cold air exposure boxes. Subjects perceived significantly less nasal congestion (greater patency), both unilaterally and bilaterally, in the cold air box and unilaterally in the dry air box, compared to the room air box (p<0.01), with the cold air box having a larger effect (p<0.01) (Wilcoxon match pairs test, with Holm–Bonferroni correction to control for multiple comparison.)

When the temperature difference between the cold and room air boxes was less than 5°C (the average difference was 11.2±3.14°C) or the RH difference between the dry and room air boxes was less than 10% RH (the average difference was 22.7±12.0%), the data points were excluded in the paired t-test but not in the correlation or regression analysis. The rationale behind the data exclusion is that the t-test examines the effect of different temperature or humidity conditions on patency sensation. If the conditions were not significantly different, due to climate changes, operational errors, or subject errors (left the cover open, breathed too long, etc.), the data point was excluded from the examination. Figures 1 and 4 reflect exclusion of four data points for the cold air box and nine data points for the dry air box. However, correlation and multiple regression analyses examine a continuous condition effect—if the air in the cold air box was not cold enough, then the analysis should predict little effect on patency for this data point—so no exclusion was necessary. Figure 6 and Table 1 are presented with no data exclusion.

**Table 1:** Significant contributors to unilateral patency ratings (forward step-wise multiple regressions).

	Regression coefficient	Partial correlation	p-Value
**Heat loss**	−0.21	−0.19	0.009321*
**Butanol log ppm**	0.14	0.15	0.047653*
**Box temperature**	0.17	0.11	0.144773

The dependent variable was unilateral nasal subjective patency; the independent variable pool consisted of exposure air temperature and humidity, nasal heat loss, butanol detection threshold, nasal resistance, and MCA (minimum airway cross-sectional area as measured by acoustic rhinometry). Criterion for a variable to enter the regression, F>1; to be removed, F<0.5; total steps = 5. Final regression model consist of two significant variables: r = 0.43, R^2^ = 0.187, adjusted R^2^ = 0.168, F(4,175) = 10.021; p<0.00001.

The study was conducted from summer through fall, while the outside air temperature and humidity varied considerably. As it turned out, our building AC system controlled room temperature better than it did room RH. However, for paired measurement and comparison, as long as the reduction of humidity in the dry air box was significantly different from that in the room air box, we met the requirements of our experimental design.

## Results

**Figure 5:**
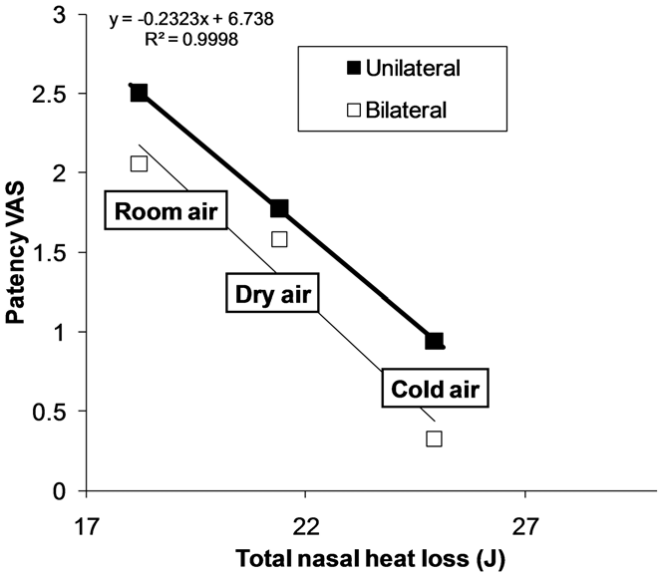
Averaged total nasal heat loss versus patency visual analog scale (VAS) ratings for the three exposure box conditions. Nasal heat loss is calculated assuming that, at the end of the breath, inspired air is always warmed and humidified to 35°C and 90% RH [13], [14]. Data are averaged over subjects for the three exposure conditions.

Subjects perceived significantly less nasal congestion (greater patency), both unilaterally and bilaterally (see Figure 4), in the cold air box and unilaterally in the dry air box, compared with the room air box (p<0.01), with the cold air box having a larger effect (p<0.01). Thus, the results confirm that air temperature is a contributing factor to the perception of nasal patency (hypothesis 1), but not the sole factor, as air humidity also contributed significantly. Total nasal heat loss, calculated by adding conductive heat loss (driven by temperature gradient) and evaporative heat loss (driven by humidity gradient) using Equation 1 (see Methods), correlated well with changes in unilateral and bilateral patency ratings (hypothesis 2; see Figure 5).

**Figure 6:**
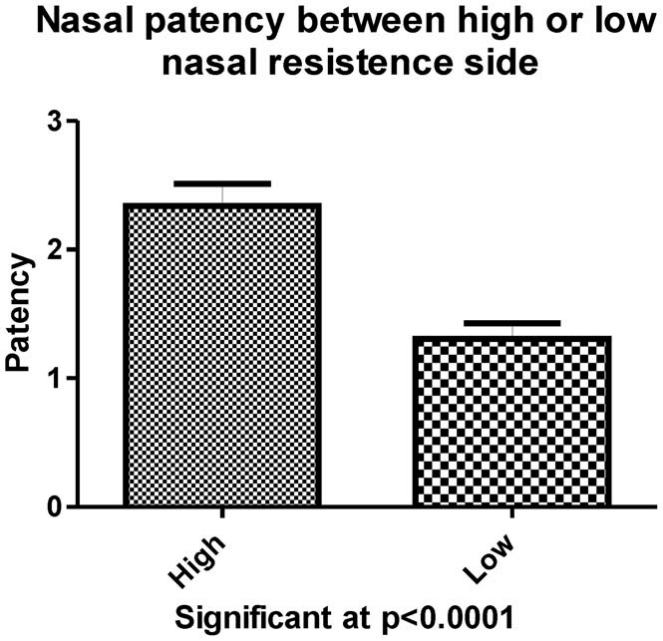
Within subject, there was a significant difference in perceived patency between the high and low resistance nostrils.

We also found significant correlations between perceived unilateral patency and exposure air temperature (Spearman rank r = 0.35, p<0.05), and between perceived unilateral nasal patency and humidity (r = 0.22, p<0.05). We found no significant correlations between perceived nasal patency and nasal resistance, MCA, or lateralization threshold to butanol (hypothesis 3). However, the correlation between nasal resistance and MCA was significant (r = 0.42, p<0.05). We also found no significant correlation between butanol lateralization threshold and either nasal resistance or MCA. We found a significant within-subject difference in perceived patency between the high- and low-resistance nostrils (Figure 6).

When combining all factors, forward stepwise multiple regression showed that nasal heat loss and butanol lateralization threshold contributed significantly to unilateral patency rating (Table 1, adjusted R^2^ = 0.168). Without the derived parameter (nasal heat loss), only temperature and humidity contributed significantly to the patency rating, although with a slightly smaller adjusted R^2^ value (R^2^ = 0.14).

## Discussions

The most interesting result of the current study is that the humidity of the inspired air, when air temperature was held constant, significantly modulated perceived unilateral patency, even though it appears that air temperature has a larger effect. In retrospect, this is because the humidity sensor reported only relative humidity—water vapor pressure as a percentage of the saturated vapor pressure—which decreases as a function of air temperature. If RH is converted to absolute humidity, the air in the cold air box was approximately as dry as that in the dry air box, which may be the reason for its stronger effect. However, the fact that humidity does contribute to patency ratings leads to the finding that nasal mucosal cooling (or heat loss) is the actual underlying stimulus in the perception of nasal patency. Water evaporation produces cooling (evaporative heat loss) as effectively as does a temperature gradient. The cold air produced greater cooling because it was both warmed and humidified while passing though the nasal airway.

This finding challenges the conventional wisdom that has often focused on static air temperature as the stimulus of patency perception. It has long been recognized that cool receptors in skin respond most strongly to dynamic temperature gradients rather than to fixed temperatures. For example, metal objects (an iron bar) are perceived as much “cooler” than, say, a wood bar, even at the same temperature, because metal has a higher heat capacity and conducts heat out of the skin much more quickly than does wood. This study confirms that the cool sensors inside the nose may function in the same way.

If we recognize perceived nasal patency as a synthesis of sensory input, it is important to understand what the actual stimuli are. The dynamic cooling (heat loss) is not just a function of the static air temperature or humidity in the environment; it also depends on the interaction between an individual's nasal airway structures and the inspired air flow. Given the same static air temperature or humidity, differences in nasal structure and physical conditions may result in different degrees of mucosal heat loss and thus lead to different experiences of nasal patency for different subjects. Wide nasal passages with the bulk of the airstream having little contact with the mucosal wall are likely to produce little mucosal cooling, similar to a constricted airway with an insufficient air stream. Nasal airway obstruction may have diverse clinical manifestations in a patient's subjective symptoms, depending on the mucosal/airflow interaction and the patient's baseline thermosensory sensitivities. Treatment of subjective obstructive symptoms may need to focus on restoring the patient's optimal mucosal/air flow cooling and healthy thermosensory functions.

**Figure 7:**
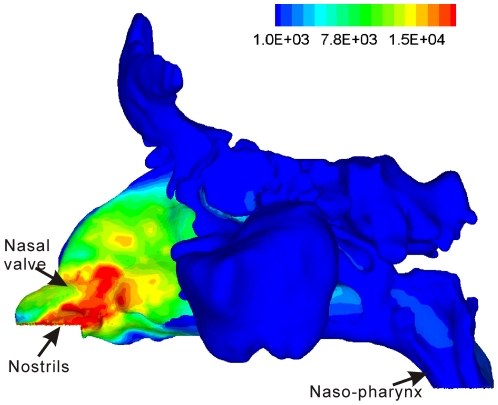
Contour plot of nasal mucosal heat flux (J/m^2^) for a subject that received a nasal/sinus CT scan immediately before testing. A CFD model was created for this subject using the method described by Zhao et al. [16] in which nasal airflow and mucosal heat exchange are then simulated. The wall boundary condition at the mucosal surface is set similar to that described by Naftali et al. [14]: fully saturated with water vapor and at body temperature, with an unlimited supply of heat and water vapor.

Regional air/mucosa contact and heat loss may be difficult, if not entirely impossible, to measure [15], but can be more easily and quantitatively simulated through computational fluid dynamics (CFD) models [14]. These CFD models are often constructed based on CT or MRI images and thus are sensitive to submucosal morphological changes (mucosal swelling or engorgement) captured by the imaging modality. Figure 7 shows the modeled mucosal heat loss gradient for one of the subjects in this study that received a nasal/sinus CT scan immediately before testing. A CFD model was created for this subject using the method described by [16], which simulated nasal airflow and mucosal heat exchange. The gradient of heat loss is not uniform but concentrates in the nasal valve and vestibule region, coinciding with reported maps of mucosal detection sensitivity to air puffs, which also peaked anteriorly [17]–[19]. This may reflect adaptive optimization to co-localize high trigeminal sensitivity to areas of greater heat loss. The combination of measured or simulated (CFD) mucosal heat loss and the measurement of mucosal sensitivity may provide potential new clinical tools (in addition to rhinometry) to decipher the symptoms associated with nasal patency, whether the disorder is related to the airway structure, to sensory factors, or to both.

Clinicians are often more interested in bilateral feedback from patients, because it reflects the natural state of breathing. However, our results were more significant for unilateral than for bilateral ratings, which is often the case in rhinological studies that attempt to relate objective measurement to subjective perceptual feedback, because the bilateral feedback is not a simple addition or average of unilateral feedback [20]. Because in the current study bilateral ratings of patency were significantly lower than unilateral ratings, possibly reflecting a central integration, we focused more on unilateral measurement (rhinometry, lateralization threshold) to explore the underlying peripheral mechanism of patency perception. However, we also included bilateral patency ratings, which may provide preliminary data for potential future clinical interpretations. Age and gender were not included as co-factors because of the limited sample size and because no strong influence of age or gender on trigeminal detection sensitivity has been found [12].

Previous studies [10] have suggested that prolonged exposure to either colder or hotter environment would change nasal volume and submucosal blood circulation, presumably due to sympathetic nervous system activation. These morphological changes would in turn modulate nasal ventilation, the pattern of air/mucosa contact, and subsequent nasal heat loss, in addition to heat loss due to air temperature or humidity changes. Potentially, this feedback loop may serve to optimize the nasal air conditioning function.

Despite the statistical significance, all the variables in this study combined account for less than 20% of the total variance in patency ratings. Many other factors may contribute both centrally and peripherally to variability in an individual's perception of patency. One possibility could be the mechanosensors in the nasal mucosa [3]. A few studies have used air puffs to investigate mechanical sensitivities in nasal mucosa [17]–[19]. Without well-controlled air temperature and humidity, air puffs also produce mucosal heat loss in addition to mechanical stimulation. Thus, drawing clear conclusions from such studies would be challenging because, from a fluid dynamics perspective, it is difficult to separate mechanical shear stress from convective or evaporative heat loss on the nasal wall during breathing. Regions with high heat exchange are also likely to have high mechanical (shear) stress. Thermo- and mechanosensitivity at the peripheral neural level can also be overlapping and synergistic [21].

Using butanol lateralization threshold to assess the general trigeminal sensitivity is common in chemosensory research, yet in this study it had no direct correlation with subjective nasal patency. However, when the heat loss contribution is teased out, the contribution of trigeminal sensitivity to nasal patency does become significant (partial correlation, stepwise regression). While trigeminal nerve endings are known to respond to innocuous cooling via activation of TRPM8 receptors, less is known about the mechanisms and neuronal subpopulations serving trigeminal responses to volatile irritants, including butanol. Volatile alcohols and aldehydes activate some but not all cool-sensitive neurons [22], which indicates the likely coexistence within some neurons of the mechanisms that respond to both cool and butanol stimulation. However, it is unlikely that the TRPM8 receptor mediates responses to butanol. Thus, the sensitivity to butanol may only partially reflect trigeminal sensitivity to heat loss. Mapping of mucosal sensitivity to menthol (similar to [23], [24]) or to well-controlled air puffs or temperature probes might yield more robust correlations with patency. However, any experimental design that involves exposure to menthol or eucalyptol (another TRPM8 ligand) must carefully address their cumulative anesthetic and desensitizing effects, the complications of which prevented us from using them in this pilot study, but they could be included in future studies.

### Conclusions

The current study confirms that the both ambient air temperature and humidity significantly modulate an individual's perception of patency through heat loss in the nasal mucosa and trigeminal sensory input. The dynamic cooling between the airstream and the mucosal wall may be quantified experimentally or computationally and could lead to new clinical evaluation tools.
